# Supplementary material for: The impact of light heterogeneity in controlled environment agriculture on biomass of microgreens
Source: Quant Plant Biol. 2025 Jul 10;6:e17. doi: 10.1017/qpb.2025.10003 (PMC12277203; doi:10.1017/qpb.2025.10003)
Supplement: Claydon et al. supplementary material [file S2632882825100039sup001.zip › SupplementalMaterials_editableFormat.docx]

**Supplemental materials for “Harnessing light heterogeneity to optimise controlled environment agriculture”**

**
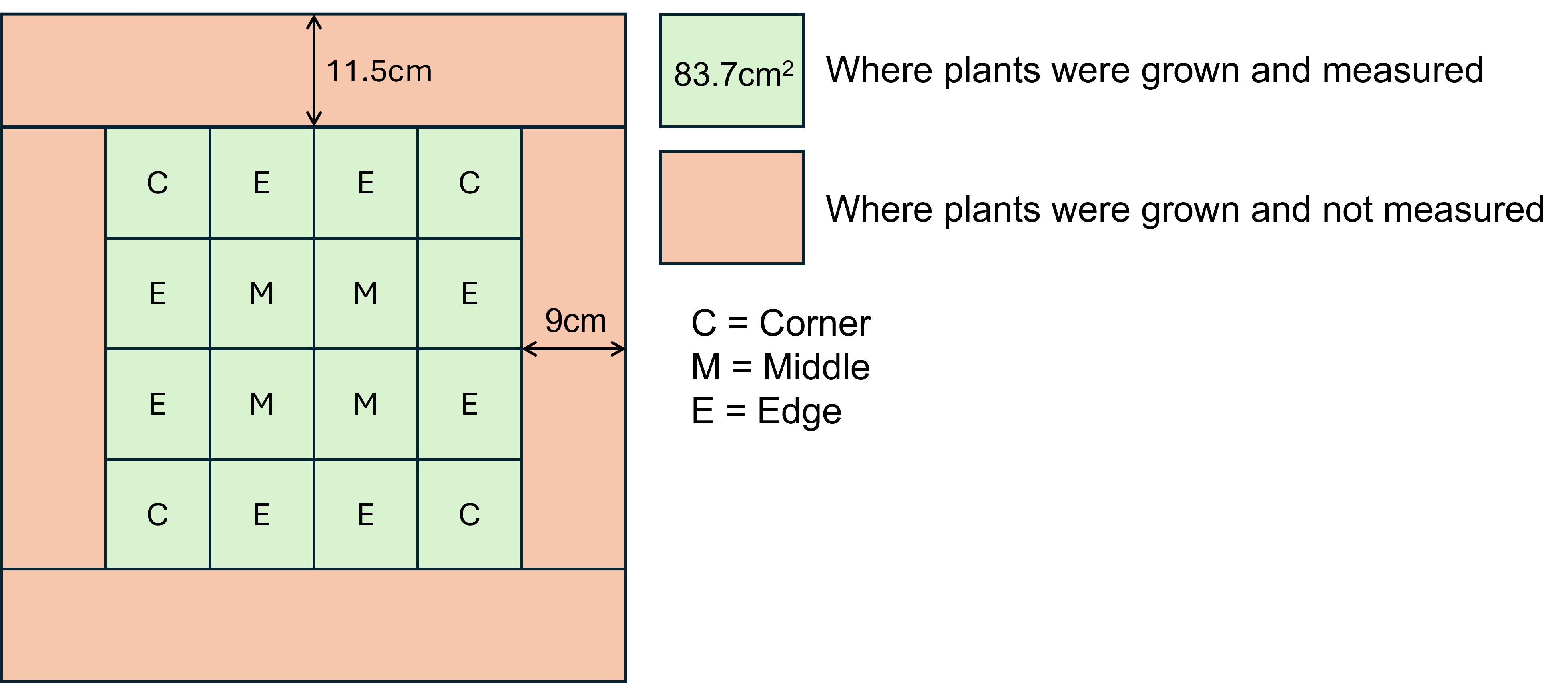
**

**Figure S1:** The layout of each tray.


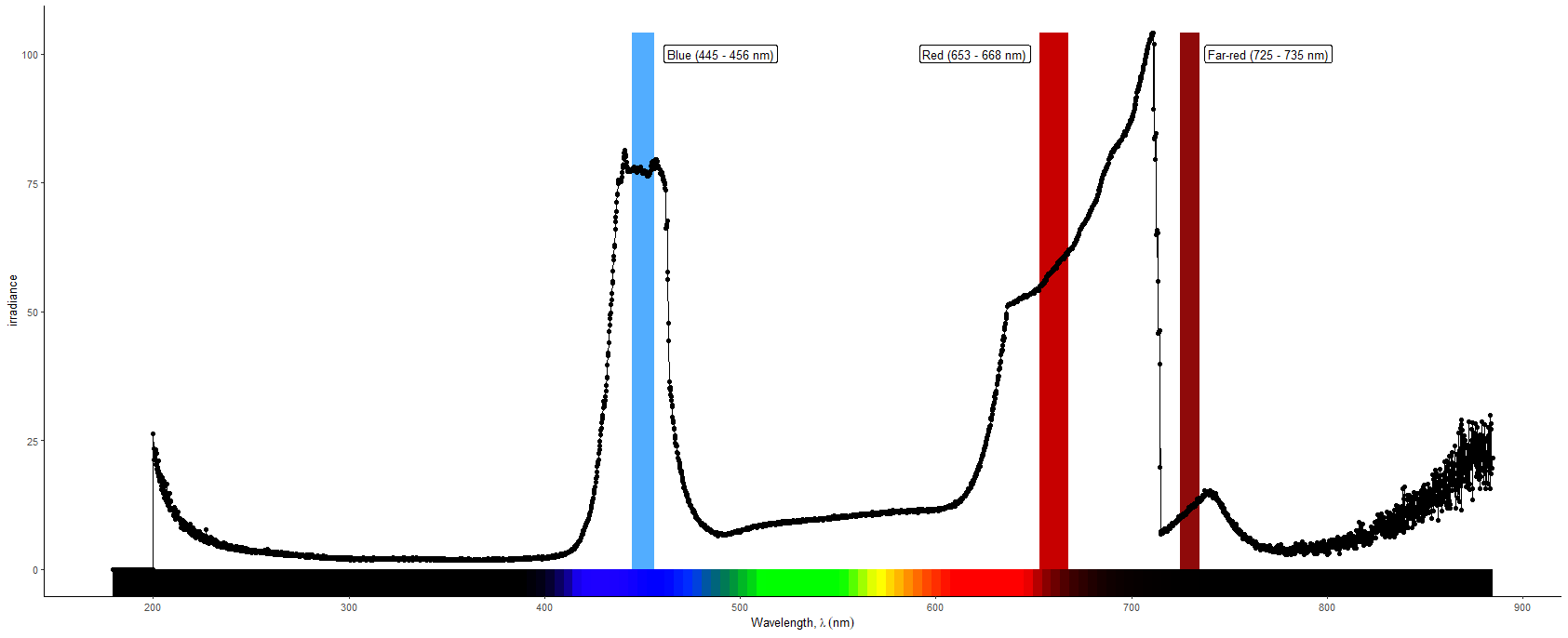


**Figure S2**: Example full light spectrum at plant level, with regions that we use to indicate blue, red, and far-red highlighted in coloured bars.


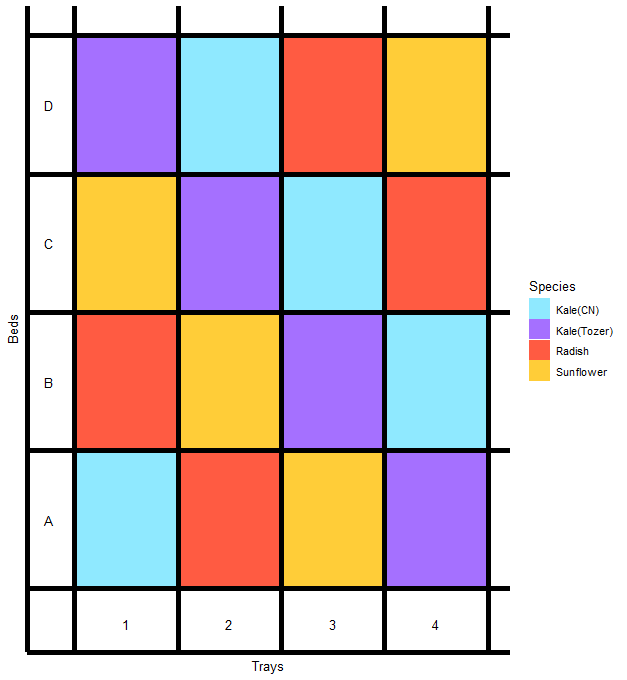


**Figure S3**: Arrangement of varieties in beds and trays.


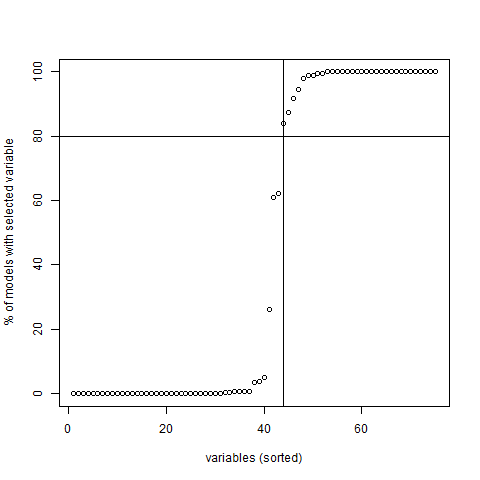


**Figure S4**: Proportion of models in which each variable was selected with non-zero coefficients.
